# Calcium-Associated Cellular Integrity and MAPK/cAMP Signaling Are Associated with Low-Temperature Acclimation in *Schizophyllum commune* 20R-7-F01

**DOI:** 10.3390/jof12100760

**Published:** 2026-10-09

**Authors:** Shuang Leng, Dongxu Li, Chen Chu, Changhong Liu

**Affiliations:** 1 State Key Laboratory of Pharmaceutical Biotechnology, School of Life Sciences, Nanjing University, Nanjing 210023, China; 2 First Clinical Medical College, Nanjing University of Chinese Medicine, Nanjing 210023, China

**Keywords:** low-temperature stress, Ca^2+^ signaling, cell wall integrity, MAPK/cAMP signaling, *Schizophyllum commune* 20R-7-F01

## Abstract

Temperature is a critical environmental factor affecting fungal growth, morphogenesis, and reproduction. *Schizophyllum commune*, a model basidiomycete fungus, exhibits remarkable developmental plasticity under environmental fluctuations; however, the mechanisms underlying its adaptation to low-temperature conditions remain poorly understood. In this study, we show that low-temperature stress inhibited mycelial growth, delayed fruiting body development and basidiospore germination, and promoted chitin accumulation and chitin synthase genes (*CHS*) expression in *S. commune* 20R-7-F01. Low-temperature exposure triggered transient oxidative stress, followed by restoration of antioxidant capacity during acclimation. Mechanistically, mitogen-activated protein kinase (MAPK) inhibition impaired mycelial growth, whereas 3′-5′-cyclic adenosine monophosphate (cAMP) content increased under low-temperature stress, suggesting the involvement of cAMP signaling in the low-temperature response. Moreover, low-temperature exposure was associated with increased mycelial calcium (Ca^2+^) content, and Ca^2+^ chelation was accompanied by impaired cell membrane integrity and altered cell wall ultrastructure. Collectively, our findings suggest that the adaptation of *S. commune* 20R-7-F01 to low temperature is associated with Ca^2+^ content, cell integrity, antioxidant defense, MAPK, and cAMP. These results provide new insights into the molecular basis of fungal low-temperature acclimation.

## 1. Introduction

*Schizophyllum commune* is a cosmopolitan wood-decaying basidiomycete fungus with a fully sequenced genome and well-established genetic resources, making it an important model organism for studying fungal development, mating systems, cell wall biology, and secondary metabolism [1,2,3]. In addition, *S. commune* produces schizophyllan, a bioactive β-glucan with pharmaceutical and industrial applications [4]. *S. commune* is one of the most commonly found fungi and can be isolated from all continents except for Antarctica [5]. The wide distribution of this species across temperate and cooler habitats suggests an ability to persist under environmentally imposed low-temperature conditions. More importantly, the *S. commune* 20R-7-F01 used in this study was isolated from deep-sea sediments, an environment characterized by extreme conditions year-round. This unique origin makes it an ideal model for investigating the molecular mechanisms of low-temperature adaptation. However, the physiological and molecular mechanisms that enable *S. commune* to adapt to low-temperature stress remain largely unknown.

As sessile organisms, fungi adapt to fluctuating environmental temperatures through complex regulatory mechanisms [6]. Low temperature is an important environmental stressor that affects fungal growth, development, and survival throughout the life cycle by restricting hyphal growth, delaying reproductive development, and influencing primordium initiation, fruiting body maturation, and spore development [7,8,9]. Our previous review systematically summarized the main strategies underlying fungal low-temperature adaptation [10]. It is important to distinguish between responses to low-temperature above freezing and mechanisms specifically related to ice formation. The former primarily involves metabolic and signaling adjustments to maintain cellular function under low-temperature conditions, whereas the latter includes antifreeze proteins and ice-recrystallization-inhibiting substances that mitigate mechanical damage caused by ice crystals. At the cellular level, low-temperature stress disrupts membrane fluidity, protein function, nucleic acid stability, and metabolic homeostasis, while also inducing the accumulation of reactive oxygen species (ROS), which can cause oxidative damage to lipids, proteins, and nucleic acids [9,11]. To mitigate these adverse effects, fungi may secrete antifreeze proteins to inhibit ice crystal growth and recrystallization, thereby reducing ice-induced mechanical damage [12,13]; accumulate osmoprotectants, such as trehalose and polyols, to lower the freezing point and maintain cellular osmotic balance [14,15]; and synthesize low-temperature shock proteins or RNA-binding proteins that function as RNA molecular chaperones to stabilize mRNA structures and facilitate translation at low temperatures [16]. In addition, fungi can maintain membrane integrity and basal metabolic activity by producing low-temperature-adapted enzymes with high catalytic efficiency at low temperature, increasing the proportion and unsaturation index of membrane fatty acids, and synthesizing glycolipid biosurfactants that possess both ice-recrystallization-inhibition and osmoprotective functions [17,18,19,20].

Moreover, antioxidant systems involving superoxide dismutase (SOD), glutathione (GSH/GSSG), and glutathione peroxidase (GSH-Px) act cooperatively to maintain cellular redox homeostasis [9], whereas cell wall stability is maintained through dynamic remodeling of chitin and glucans, enhanced chitin synthesis, and the regulation of chitin synthase (*CHS*) genes [21,22]. The accumulation of protective substances such as melanin and extracellular polymeric substances (EPS), together with ecological avoidance strategies such as the timing of spore germination to warmer seasons, may further enhance fungal low-temperature tolerance [23,24]. These protective mechanisms are generally associated with conserved signaling networks, including the mitogen-activated protein kinase (MAPK), 3′-5′-cyclic adenosine monophosphate (cAMP), and Ca^2+^ signaling pathways [9,25,26,27,28,29,30]. Among these, the cAMP, MAPK, and Ca^2+^ signaling pathways, together with cellular integrity and antioxidant responses, represent the primary processes hypothesized to operate at 10 °C in this study. Although studies in other fungi have identified diverse mechanisms that mitigate low-temperature-induced damage, whether these responses operate in *S. commune* 20R-7-F01, and how they are associated with its low-temperature adaptation, remain unclear.

In this study, we investigated the physiological and molecular responses of *S. commune* 20R-7-F01 to low-temperature stress. We hypothesize that this deep-sea-derived strain possesses unique low-temperature mechanisms, and that its physiological and molecular responses to low temperature are associated with MAPK, cAMP, and Ca^2+^ signaling pathways. To test this hypothesis, we first performed a systematic physiological characterization of low-temperature responses, including the effects of low-temperature exposure on mycelial growth, fruiting body development, and basidiospore germination, as well as low-temperature-induced changes in chitin content and *CHS* gene expression, oxidative-stress biomarkers, and antioxidant capacity. On this basis, we further conducted an exploratory investigation of the signaling pathways potentially involved in low-temperature adaptation using transcript analysis and pharmacological treatments, with a focus on MAPK, cAMP, and Ca^2+^ associated signaling responses. This study elucidates the potential mechanisms by which the *S. commune* 20R-7-F01 responds to low-temperature stress. Unlike previous studies that primarily focused on the low-temperature response of mesophilic or terrestrial fungi, this research offers a unique perspective by linking the low-temperature adaptation strategies of a deep-sea-derived basidiomycete to its distinctive ecological origin, thereby deepening our understanding of environmental adaptation in basidiomycetes.

## 2. Materials and Methods

### 2.1. Fungal Strain

The strain *Schizophyllum commune* 20R-7-F01 (CGMCC 5.2202) used in this study was isolated from IODP Expedition 337 [31]. Detailed information about the habitat, minimal medium (MM, 20 g C_6_H_12_O_6_, 1.5 g C_4_H_8_N_2_O_3_, 1.0 g K_2_HPO_4_, 0.46 g KH_2_PO_4_, 0.5 g MgSO_4_·7H_2_O, 0.12 mg Vitamin B1, 5 mg FeC1_3_·6H_2_O, and trace elements in 1 L of deionized water), and culture conditions for this strain (dikaryotic strain) have been previously described [32,33]. Unless otherwise indicated, all chemicals and reagents used in this study, including those for MM preparation and experimental treatments, were purchased from Sigma-Aldrich (St. Louis, MO, USA). This strain was selected because it is an established model strain in our laboratory and has been widely used in our previous research; furthermore, its well-defined genetic background and comprehensive genomic information make it highly suitable for this study.

### 2.2. Experimental Treatment

Preculturing involved inoculating a 2 mm diameter mycelial plug onto solid MM and incubating it at 30 °C for 96 h. For solid-state culture experiments, the growth of *S. commune* 20R-7-F01 was evaluated by inoculating 2 mm diameter mycelial blocks onto solid MM and culturing them at different temperatures (5, 10, 15, 20, 25 and 30 °C) for 96 h [34].

For experiments in liquid culture medium, fresh mycelium was collected from the growing edge of the colony, homogenized, and inoculated into 500 mL Erlenmeyer flasks containing 200 mL of MM; incubation was carried out at 30 °C with agitation at 200 rpm for 72 h. The mycelium was filtered through gauze and washed 3 times with sterile deionized water; the resulting mycelium served as the inoculum for the experiments.

Subsequently, 1 g (wet weight) of inoculum was transferred into a 250 mL Erlenmeyer flask containing 150 mL of MM, either with or without the addition of 10 μM U0126 [35] or 10 mM EDTA (Ca^2+^ chelating agent) [36] or 20 μM BAPTA-AM (a membrane-permeable, Ca^2+^ specific chelator) [37]. U0126, a MEK inhibitor commonly used to target ERK-type MAPK signaling in other systems, was used as a pharmacological treatment in this study. Its molecular target and the effect on mitogen-activated protein kinase (MAPK) phosphorylation were not directly assessed in *S. commune* 20R-7-F01. EDTA was directly dissolved in the liquid MM to a final concentration of 10 mM, while BAPTA-AM was first dissolved in DMSO as a stock solution and then added to the liquid MM to a final concentration of 20 μM (with a final DMSO concentration of ≤0.1%, *v*/*v*). An equivalent volume of DMSO was added to the control group to exclude solvent effects. The dissolution method for U0126 is the same as that for BAPTA-AM. All treatments were incubated with shaking at 200 rpm at 5–30 °C for 96 h. After the incubation period, mycelial growth and related parameters were measured.

### 2.3. Determination of Relative Growth

The diameter method was employed. Upon completion of cultivation, colony diameters were measured from different directions, and the average value was calculated. Relative growth was determined based on net growth after subtracting the initial diameter of the mycelial plug; the formula used was: Relative growth (%) = (D_t_ − D_0_)/(D_k_ − D_0_) × 100%, where D_t_ represents the colony diameter of the treatment group at time t, D_0_ is the initial diameter of the mycelial plug (2 mm), and D_k_ is the colony diameter of the control group at the same time point.

### 2.4. Determination of Biomass

Upon completion of cultivation, the mycelia were separated from the culture medium by filtration through gauze and washed three times with sterile deionized water. The mycelia were wrapped in pre-dried and pre-weighed filter paper and then dried in a constant-temperature oven at 60 °C for 72 h. After drying, the biomass (dry weight) was calculated by weighing the sample on an analytical balance and subtracting the initial weight of the filter paper. The net biomass accumulation was determined by subtracting the initial biomass measured using the same procedure prior to cultivation from the final biomass. For fresh weight determination, surface moisture was blotted off with filter paper immediately after filtration, and the sample was weighed right away.

### 2.5. Basidiospore and Fruiting Body Culture

Basidiospore germination: Collected basidiospores (on the fruiting bodies of *S. commune* 20R-7-F01) were suspended in sterile deionized water at a concentration of 1 × 10^8^ mL^−1^. Then, 1 mL of the basidiospore suspension was transferred to a culture flask containing 15 mL of liquid MM medium (the basidiospore concentration at this point was 10^6^ mL^−1^) [33]. The suspension was incubated at 10 or 30 °C for a specified time, and the basidiospore length was measured. The germination process of basidiospores involves several stages: activation, isotropic growth, and polarized growth. Initially, basidiospores undergo activation (during which their length remains virtually unchanged), triggering the germination process. This is followed by isotropic growth, which is marked by swelling and water uptake, along with wall growth (during which the basidiospore length increases slowly). Polarized growth begins once wall deposition becomes polarized, forming a germ tube (at which point the basidiospore length increases significantly) [38]. In this study, a basidiospore was considered germinated when a visible germ tube emerged, marking the onset of polarized growth. During the germination of basidiospores, observations were made at 2 h intervals; at each time point, 20 basidiospores were randomly examined under an Olympus BX53 light microscope (Olympus, Hachioji, Tokyo, Japan), and the length of each individual basidiospore was measured.

Fruiting body culture: To assess fruiting body development and gene expression associated with fruiting body formation, mycelial blocks with a diameter of 2 mm were cultured on solid MM medium at 10 °C and 30 °C for 168 h. The cultures were maintained at their respective temperatures and then exposed to a 600 h light treatment (12 h day/12 h night). Throughout the entire cultivation process, all cultures were kept in an artificial climate chamber (MGC-450HP, Shanghai Yiheng Scientific Instrument Co., Ltd., Shanghai, China) to ensure stable temperature and light conditions.

After the mycelium shifts from vegetative to reproductive growth, the small, dark brown protrusions visible to the naked eye on the surface of the substrate are defined as primordia; the time of their initial appearance serves as the recording point. Further differentiation of the primordia into young fruiting bodies characterized by distinct stipe and cap structures marks the stage of fruiting body formation; the time when the differentiation of the cap and stipe is complete and the fruiting body’s outline is clearly discernible is used as the recording point for this stage. Fruiting body maturity is reached when the fruiting body develops fully, the cap expands completely with flat edges, and spore release has either not yet begun or is just commencing; the time of complete cap expansion serves as the recording point for this stage. Observations are conducted every 6 h following the start of the experiment, with detailed records kept of the time required to reach each developmental stage.

### 2.6. Chitin Content

Chitin content was quantitatively determined using the glycosaminoglycan glucosamine method [39]. The cultured mycelia were washed three times with sterile deionized water to remove residual culture medium components. Subsequently, the collected mycelia were blotted dry with sterile absorbent paper, and an accurately weighed 0.1 g sample was placed in 6 mL of 0.5 mol L^−1^ hydrochloric acid solution for hydrolysis (110 °C, 2 h). The hydrolysate was then subjected to a colorimetric reaction, and the absorbance was measured at 650 nm. Absorbance was recorded using an Infinite M200 Pro microplate reader (Tecan, Research Triangle Park, Durham, NC, USA). A standard curve was constructed using D-glucosamine hydrochloride as the standard (*r*^2^ > 0.99), and the content was calculated using the formula C × V × D × W^−1^ and expressed in μg g^−1^ FW. C: concentration of glucosamine calculated from the standard curve (μg mL^−1^); V: total volume of the reaction system (mL); D: sample dilution factor; W: fresh weight of the mycelial sample used for hydrolysis (g).

### 2.7. Determination of Oxidation and Antioxidation Related Biochemical Indicators

Commercial kits (Solarbio, Beijing, China) were used to measure redox-related biochemical parameters, including reactive oxygen species (ROS, Cat^#^ D6470), malondialdehyde (MDA, Cat^#^ BC0025), reduced glutathione (GSH, Cat^#^ BC1175), oxidized glutathione (GSSG, Cat^#^ BC1185), total antioxidant capacity (T-AOC, Cat^#^ BC1310), glutathione peroxidase (GSH-Px, Cat^#^ BC1190), and superoxide dismutase (SOD, Cat^#^ BC0170). The specific experimental procedures were performed strictly according to the manufacturer’s instructions. ROS content was determined using DCFH-DA (2’,7’-dichlorodihydrofluorescein diacetate) [40]. MDA determination involved the reaction of MDA with thiobarbituric acid (TBA) to form MDA-TBA2 (maximum absorbance at 535 nm) [41]. The assays for GSH, GSSG, and GSH-Px were based on the principle that GSH reacts with 5,5’-dithiobis-2-nitrobenzoic acid to produce 2-nitro-5-thiobenzoic acid (maximum absorbance at 412 nm) [42,43]. T-AOC detection was based on the reduction of Fe^3+^-TPTZ to blue Fe^2+^-TPTZ (maximum absorbance at 593 nm) by antioxidant substances in an acidic environment [44]. The principle of SOD detection is that SOD inhibits the reduction of nitroblue tetrazolium (NBT) by superoxide anions (O^2−^) to form blue formazan (maximum absorbance at 560 nm) [45]. Protein concentration was measured using the Bradford method [46]. 

### 2.8. Determination of Ca^2+^ Content

Following experimental treatment, samples were washed three times with sterile deionized water to remove residual medium. After blotting dry with sterile absorbent paper, a 0.1 g portion was accurately weighed into a digestion tube; 5 mL of concentrated nitric acid (guaranteed reagent grade) was added, and the mixture was thoroughly homogenized and incubated at 25 °C for 168 h. Upon completion of digestion, the sample was diluted to 10 mL with 1% LaCl_3_ solution, mixed well, and filtered through a 0.22 μm membrane (Merck, Darmstadt, Germany). The Ca^2+^ content was determined using inductively coupled plasma atomic emission spectrometry (ICP-AES; PS-1000, Leeman Labs, Hudson, NH, USA). The Ca^2+^ content was calculated using the following formula: C × V × D × W^−1^ and expressed in μg g^−1^ FW. Here, C represents the Ca^2+^ concentration measured by ICP-AES (μg mL^−1^), V is the final volume (10 mL), D is the dilution factor (1), and W is the fresh weight of the mycelia (g).

### 2.9. Gene Expression Analysis Using RT-qPCR

Gene expression levels were assessed using reverse transcription quantitative polymerase chain reaction (RT-qPCR) [47]. Total RNA was extracted from 100 mg of mycelia or basidiospores, which were harvested by centrifugation, using RNAiso Plus (TaKaRa, Dalian, China) according to the manufacturer’s protocol. RNA quantity and quality were determined using a NanoDrop 2000 spectrophotometer (Thermo Fisher Scientific, Waltham, MA, USA), with A260/A280 ratios of 1.8–2.0 and A260/A230 *>* 2.0, and RNA integrity was verified by 1% agarose gel electrophoresis. One microgram of total RNA was used for reverse transcription. Reverse transcription was performed using the PrimeScript RT Reagent Kit, which contains a gDNA Eraser (TaKaRa), in strict accordance with the provided instructions. Quantitative PCR analysis of the target genes was carried out using SYBR Premix Ex Taq II (TaKaRa) on a QuantStudio 5 Real-Time PCR System (Applied Biosystems, Thermo Fisher Scientific, Waltham, MA, USA). Each 20 μL reaction contained 10 μL of 2 × SYBR Premix Ex Taq II, 1 μL of cDNA, 0.4 μM each of forward and reverse primers, 0.4 μL of ROX reference dye, and nuclease-free water to volume. The thermal cycle for RT-qPCR amplification was as follows: 95 °C for 30 s, followed by 43 cycles of 95 °C for 10 s, 58.5 °C for 30 s, and 72 °C for 30 s, with a final melting curve analysis from 65 °C to 95 °C. No-template controls (NTCs) and no-reverse-transcription controls (NRTs) were included as negative controls. Primer specificity was confirmed by a single peak in the melting curve and a single band on agarose gel electrophoresis. Amplification efficiency was assessed using a 10-fold cDNA dilution series, with slopes between 3.1 and 3.6, efficiency between 90% and 110%, and *r*^2^ > 0.99. Relative gene expression was calculated using the 2^−ΔΔCT^ method [48], with *Actin* as the reference gene. The primers used for gene expression quantification are detailed in Supplementary Table S1. All primers were synthesized by Sangon Biotech (Shanghai, China).

### 2.10. Determination of cAMP Content

1 g (fresh weight) sample of mycelia was washed three times with sterile deionized water, ground into a powder using liquid nitrogen, and mixed with 4 mL of sterile deionized water; extraction was performed in a water bath at 60 °C for 48 h. Subsequently, the mixture was centrifuged at 14,000× *g* and 4 °C for 10 min, and the supernatant was collected and adjusted to a final volume of 5 mL. The extract was filtered through a 0.22 μm microporous membrane prior to analysis. The analysis was performed using an ACQUITY UPLC H-Class System (Waters corporation, Milford, MA, USA) equipped with an ACQUITY UPLC HSS T3 column (2.1 × 100 mm, 1.8 μm; Waters corporation, Milford, MA, USA). The mobile phase consisted of a 0.02 mol L^−1^ KH_2_PO_4_ solution and methanol (96:4, *v*/*v*) at pH 2.38. The column temperature was set to 30 °C, and the detection wavelength was 254 nm. The flow rate was 0.2 mL min^−1^, and the injection volume was 5 μL [37].

### 2.11. Evans Blue Staining

Fungal mycelia treated with different methods were collected and washed three times with sterile distilled water to remove residual culture medium. The samples were then incubated in 0.05% (*w*/*v*) Evans blue solution at room temperature in the dark for 5 min [49]. Evans blue staining was performed to evaluate the integrity of the fungal cell membrane, with increased dye uptake serving as an indicator of membrane damage or loss of membrane integrity. After staining, the mycelia were thoroughly washed with sterile distilled water until no further significant dye release was observed in the tissue. Finally, the stained mycelia were observed using an optical microscope. For microscopy, samples were examined under an Olympus BX53 microscope with a DP80 camera. Images were captured in bright-field mode at 400 × magnification using identical settings across all groups. At least five random fields per biological replicate (three replicates per group, ≥15 fields total) were imaged. Images with clear focus, uniform mycelial distribution, and no impurities were selected to present the experimental results. Controls included untreated mycelia at 10 °C and 30 °C, EDTA-treated, BAPTA-AM-treated, and DMSO solvent control.

### 2.12. Transmission Electron Microscopy (TEM) Observation of Cell Walls

Fungal samples fixed with glutaraldehyde were rinsed with phosphate-buffered saline (PBS, dissolve 2.6 g of NaH_2_PO_4_·H_2_O and 29 g of Na_2_HPO_4_·12H_2_O in 400 mL of sterile deionized water, adjust the pH to 7.4, and make up the volume to 500 mL) and then post-fixed in 1% osmium tetroxide at 4 °C for 4 h. The samples were dehydrated using a graded ethanol series, embedded in epoxy resin, and sectioned into ultrathin slices (50–100 nm thick). The sections were double-stained with a saturated 2% uranyl acetate solution and lead citrate, mounted on copper grids, dried, and examined using a Talos L120C G2 transmission electron microscope (Thermo Fisher Scientific, Waltham, MA, USA). During observation, focus and astigmatism were adjusted at high accelerating voltages (80–120 kV), and appropriate regions were selected for bright-field imaging. Three independent biological samples were processed for each treatment group. Five intact mycelial cross-sections were randomly selected from each sample for observation. The images shown are representative of the observed morphological characteristics.

### 2.13. Statistical Analysis

All the experiments were conducted with a minimum of three biological replicates. Data are presented as the mean ± standard error. Statistical analysis was performed using SPSS version 28.0.1.1 (IBM Corp., Armonk, NY, USA) and ImageJ 2.14.0 software (National Institutes of Health, Bethesda, MD, USA). Normality was assessed using the Shapiro–Wilk test, and homogeneity of variance was assessed using Levene’s test. For comparisons among multiple groups, one-way or two-way analysis of variance (ANOVA) was used as appropriate. A two-way ANOVA (temperature × time) was used to evaluate the main effects of temperature and time and their interaction. When the interaction was not significant, main effects were interpreted; when the interaction was significant, simple effects analysis was performed to compare 10 °C and 30 °C at each time point (24 h and 96 h). Post hoc pairwise comparisons were performed using Tukey’s HSD test when variances were homogeneous and Games-Howell test when variances were unequal. The significance level for all statistical analyses was set at *p <* 0.05, *p <* 0.01 and *p <* 0.001.

## 3. Results

### 3.1. Low-Temperature Inhibits Mycelial Growth

**Figure 1:**
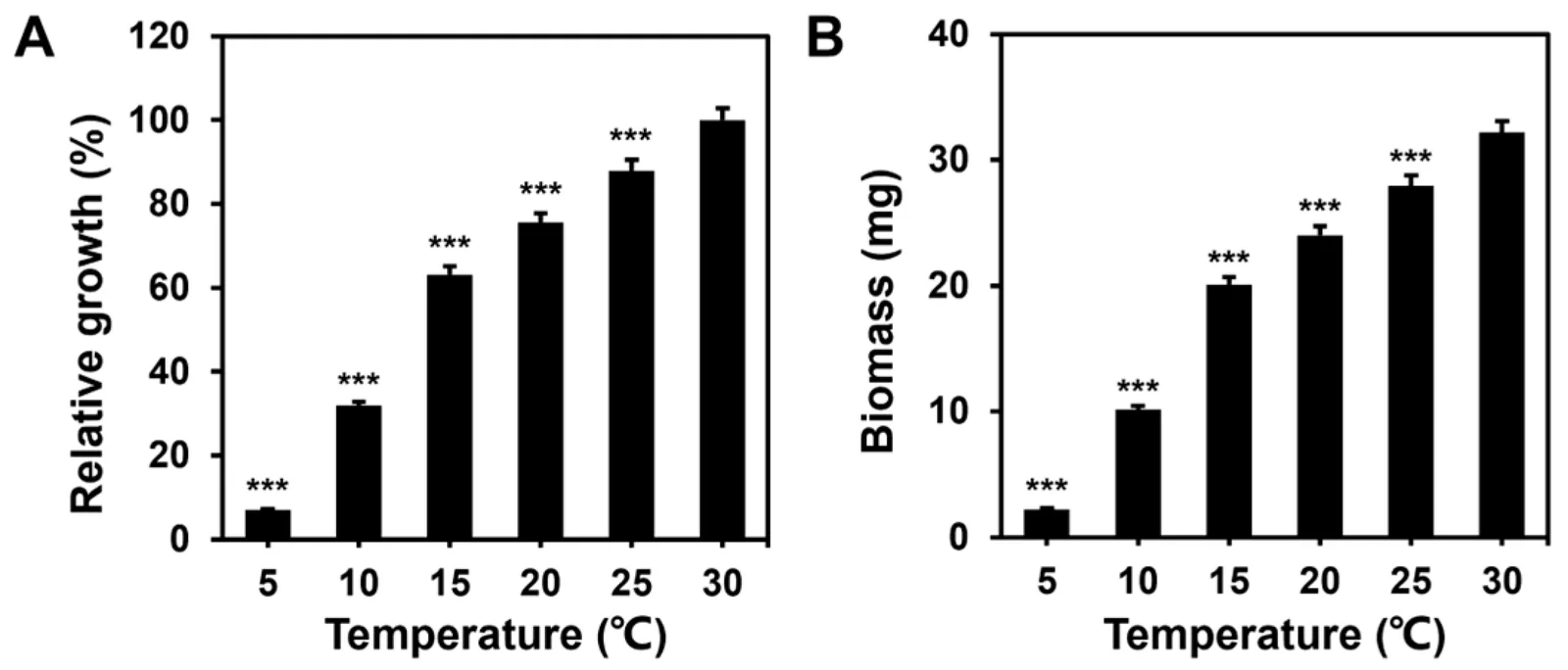
Low temperature inhibits the growth rate (**A**), and biomass (**B**) of *S. commune* 20R-7-F01. Mycelia were grown on solid (**A**) or liquid (**B**) MM. Relative growth rate is expressed relative to the mycelial growth rate at 30 °C. All treatment groups were cultured for 96 h. Values are presented as mean ± standard error (*n* = 20). Asterisk (*) indicates a significant difference compared to the 30 °C culture group. *** *p* < 0.001.

To investigate the effect of low temperature on mycelial growth, mycelial plugs (2 mm in diameter) were inoculated onto solid MM medium and cultured for 96 h. Compared with the optimal growth temperature (30 °C), colony growth was reduced by 12.08%, 24.42%, 36.83%, 68.06% and 93.00% at 25 °C, 20 °C, 15 °C, 10 °C, and 5 °C, respectively (Figure 1A). Similarly, when cultured in liquid MM medium under the same conditions, biomass was observed to decrease as the temperature dropped (Figure 1B). These results indicate that low temperature inhibits mycelial growth in a temperature-dependent manner by suppressing the polar extension of hyphae. At 10 °C, a distinct low-temperature phenotype is produced without completely inhibiting mycelial growth; therefore, 10 °C was selected as the temperature for subsequent low-temperature experiments.

### 3.2. Low-Temperature Delayed Fruiting Body Development and Basidiospore Germination

**Figure 2:**
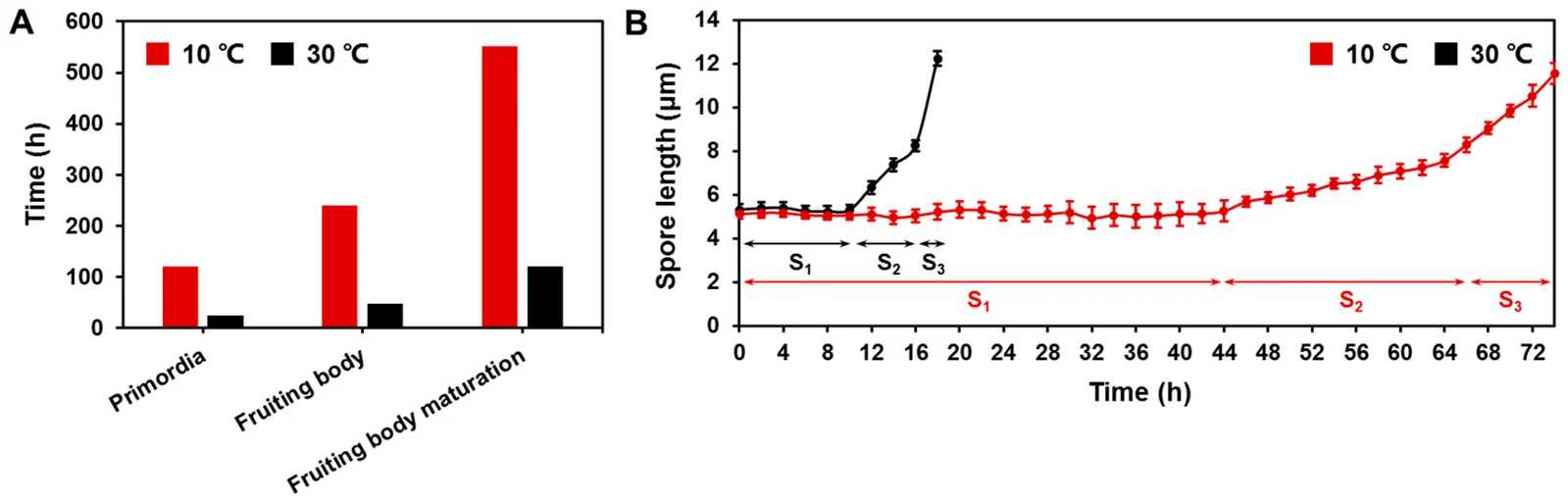
Time required for fruiting body primordium initiation, fruiting body emergence, and fruiting body maturation under different temperature conditions. (**A**) Time required for primordium initiation, fruiting body emergence, and fruiting body maturation at 10 °C and 30 °C. Representative developmental timeline. (**B**) S_1_, S_2_, and S_3_ represent the basidiospore activation, isotropic growth, and polarized growth stages, respectively. Values are presented as mean ± S.E. (*n* = 20).

The effects of low temperature on fruiting body and basidiospore development were evaluated by monitoring the timing of each developmental stage. Compared to the 30 °C condition, low-temperature treatment significantly delayed the emergence of fruiting body primordia, as well as the formation and maturation of the fruiting bodies (Figure 2A). Similarly, under low-temperature conditions (10 °C), the basidiospore growth was slower than at 30 °C. This was reflected in a lag in spore elongation, with the germination time delayed from 18 h to 74 h (Figure 2B). These results indicate that low-temperature delays both fruiting body development and basidiospore germination, thereby slowing down the overall reproductive process.

### 3.3. Low-Temperature Stress-Induced Cell Wall Remodeling

**Figure 3:**
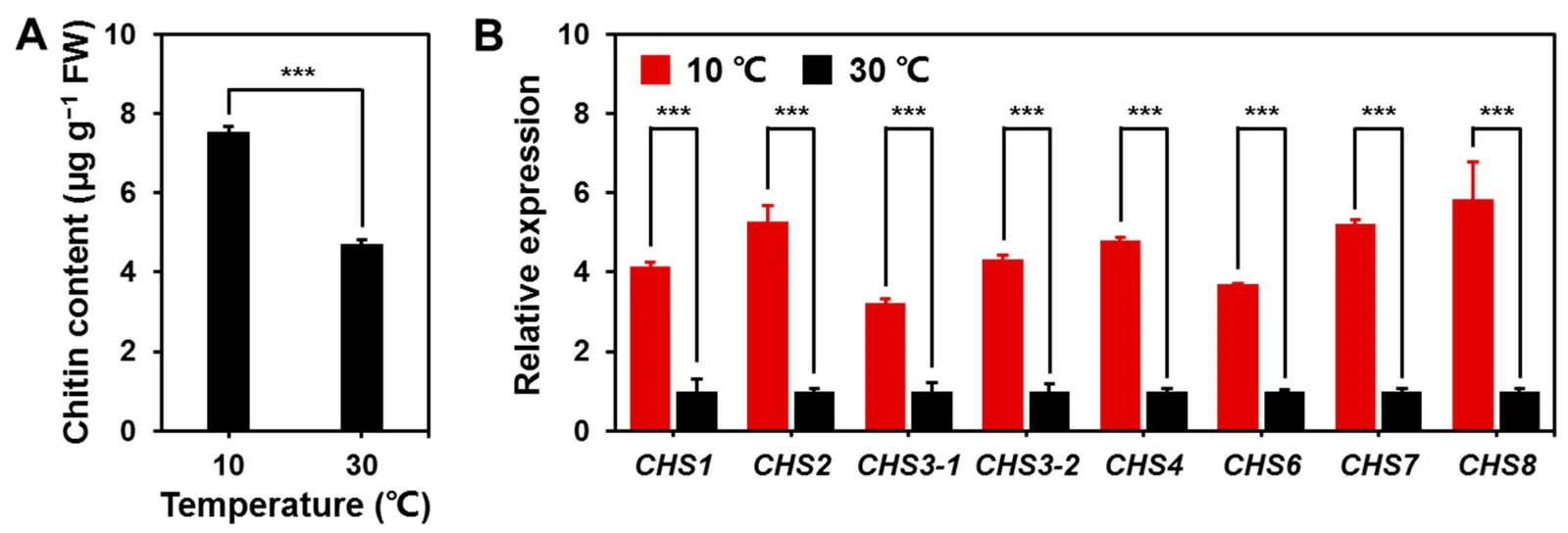
Low-temperature stress induces chitin accumulation and transcriptional activation of chitin synthase genes. (**A**) Chitin content in mycelia cultured at different temperatures. (**B**) Relative expression levels of genes encoding chitin synthase (CHS), determined by RT-qPCR. Genes associated with CHS: *CHS1*, *CHS2*, *CHS3-1*, *CHS3-2*, *CHS4*, *CHS6*, *CHS7*, and *CHS8*. Mycelia were cultured in 150 mL of liquid MM at 30 °C or 10 °C for 96 h. μg g^−1^ FW represents the chitin content per g of fresh weight. Data are expressed as mean ± S.E (*n* = 3). Asterisk (*) indicates a significant difference compared to the 30 °C culture group. *** *p* < 0.001.

Low-temperature stress altered cell wall composition by promoting chitin accumulation. Compared to the control group (30 °C), chitin content increased significantly by 59.42% following low-temperature treatment (10 °C) (Figure 3A). Consistent with this change, genes encoding chitin synthase (*CHS*) were upregulated under low-temperature stress, although induction levels varied among the different genes (Figure 3B). Notably, the transcript levels of *CHS* genes were markedly higher than those in the control group. These results indicate that low-temperature stress is accompanied by chitin accumulation and increased *CHS* transcript levels, suggesting that cell wall integrity may be threatened. However, it should be noted that the upregulation of *CHS* transcripts does not necessarily reflect a corresponding increase in chitin synthesis rate, as chitin content is determined by the balance between synthesis and degradation. Therefore, these data should be interpreted as evidence of transcriptional activation and chitin accumulation rather than a direct measure of chitin biosynthesis rate.

### 3.4. Low-Temperature Activated Antioxidant and Protective Responses

**Figure 4:**
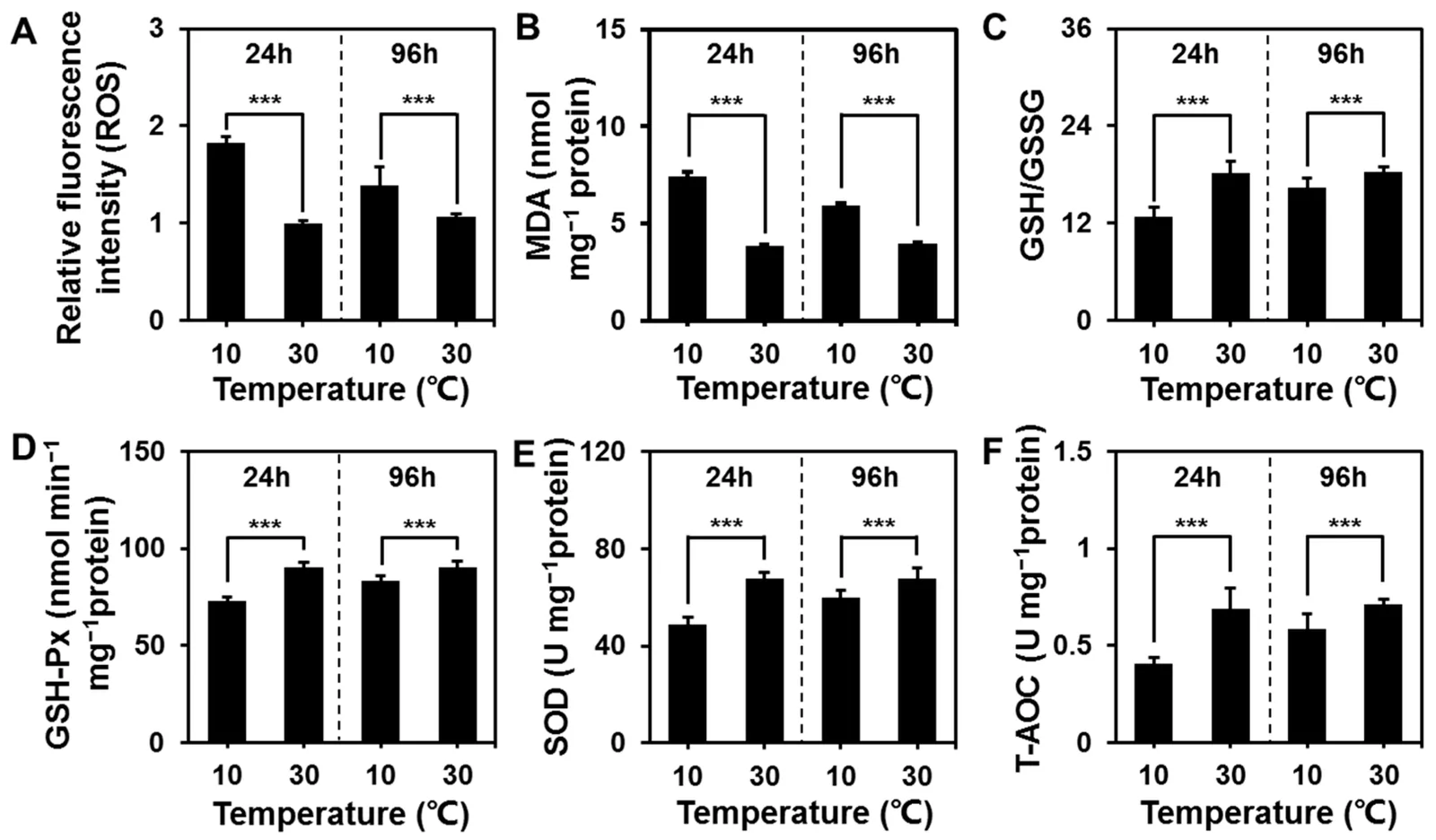
Dynamic changes in oxidative stress markers of *S. commune* 20R-7-F01 after culturing at 30 °C and 10 °C for 24 h and 96 h. (**A**) Reactive oxygen species (ROS) accumulation. (**B**) Malondialdehyde (MDA) content, the end product of lipid peroxidation. (**C**) Glutathione (GSH/GSSG) activity, and the ratio of reduced glutathione to oxidized glutathione (GSH/GSSG). (**D**) Glutathione peroxidase (GSH-Px) activity. (**E**) Superoxide dismutase (SOD) activity. (**F**) Total antioxidant capacity of cells (T-AOC). nmol mg^−1^ protein represents the content per milligram of protein. U mg^−1^ protein represents the enzyme activity per mg of protein. nmol min^−1^ mg^−1^ protein represents the amount of substrate converted per milligram of protein per minute, which is the specific activity of the enzyme. Mycelia were cultured in 150 mL of liquid MM medium at 10 °C and 30 °C for 24 or 96 h. Data are presented as mean ± S.E (*n* = 3). Asterisk (*) indicates a significant difference compared to the 30 °C culture group. *** *p* < 0.001.

To characterize the oxidative stress response of *S. commune* 20R-7-F01 under low-temperature stress, we measured six oxidative biomarkers (Figure 4A–F). After 24 h of low-temperature treatment, the levels of intracellular reactive oxygen species (ROS) and the content of malondialdehyde (MDA), the final product of lipid peroxidation, were significantly higher than those in the 30 °C treatment group (Figure 4A,B). Correspondingly, the contents of antioxidant components, such as glutathione (GSH/GSSG), glutathione peroxidase (GSH-Px), superoxide dismutase (SOD), and total antioxidant capacity (T-AOC), decreased (Figure 4C–F).

After 96 h of low-temperature culture, the accumulation of ROS and MDA in the 10 °C group was alleviated (Figure 4A,B). At the same time, GSH/GSSG, GSH-Px, SOD, and T-AOC all recovered to levels much higher than those at 24 h of low-temperature treatment (Figure 4C–F), although some indicators were still slightly lower than those in the 30 °C control group at the same time point. Collectively, short-term low-temperature stress induced severe oxidative damage in *S. commune* 20R-7-F01, whereas the fungus could gradually restore antioxidant capacity and rebalance redox status during long-term low-temperature acclimation.

### 3.5. MAPK Signaling Pathway Affects Low-Temperature-Responsive Mycelial Growth

**Figure 5:**
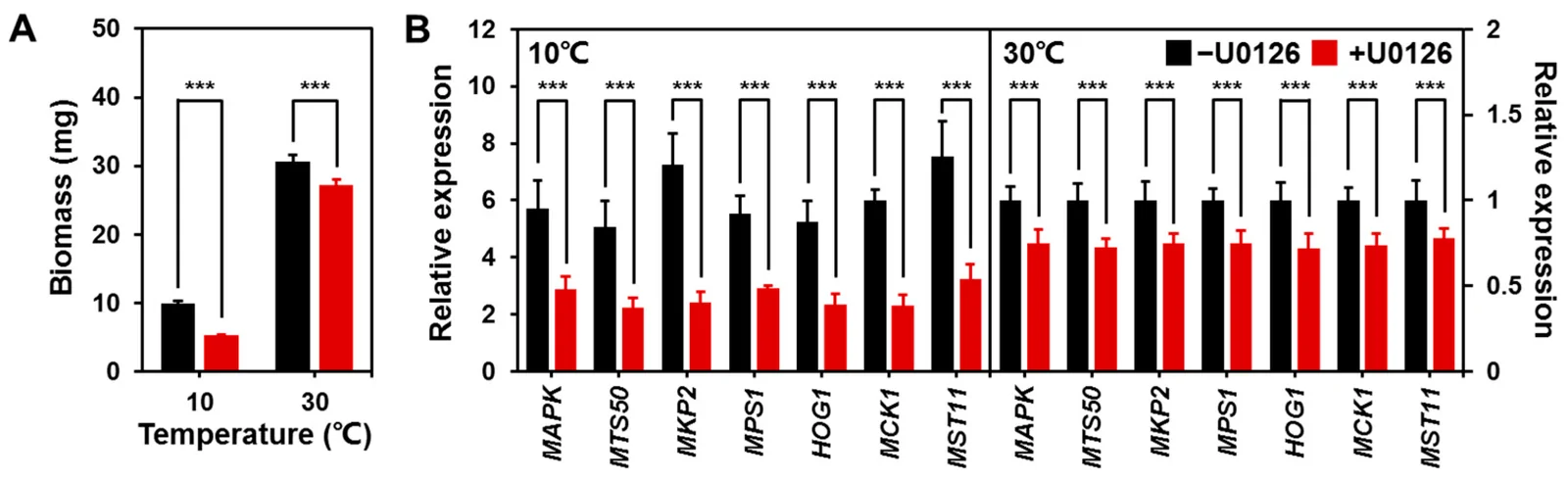
Inhibition of MAPK signaling suppresses mycelial growth and transcription of MAPK cascade genes in *S. commune* 20R-7-F01 under low temperature. (**A**) Mycelial biomass of the untreated group and the U0126 treatment group. (**B**) Relative expression levels of seven core MAPK pathway genes. Mycelia were cultured in 150 mL of liquid MM with or without 10 μM U0126 at 10 °C and 30 °C for 96 h. Values are presented as mean ± S.E. ((**A**) *n* = 20; (**B**) *n* = 3). Asterisk (*) indicates a significant difference compared to the group cultured without U0126. *** *p* < 0.001.

To verify the regulatory function of the MAPK signaling pathway in the low-temperature adaptation of *S. commune* 20R-7-F01, we treated mycelia with the MAPK inhibitor U0126 under low-temperature conditions (10 °C) and detected fungal biomass and transcriptional levels of core MAPK pathway genes. Biomass analysis showed that the mycelial biomass of the U0126-treated group was significantly lower than that of the untreated control group (Figure 5A), indicating that inhibition of the MAPK signaling pathway significantly inhibits the vegetative growth of *S. commune* 20R-7-F01 under low-temperature stress. In addition, the biomass of the U0126-treated group at 30 °C was also lower than that of the untreated group.

RT-qPCR results showed that, compared with the control, the relative expression levels of all detected MAPK signaling pathway genes (*MAPK*, *MTS50*, *MKP2*, *MPS1*, *HOG1*, *MCK1*, *MST11*) were significantly downregulated after U0126 treatment (Figure 5B). In contrast, at 30 °C, the relative expression levels of all genes were generally low, yet U0126 treatment still significantly downregulated the expression of all genes examined (Figure 5B). In conclusion, chemical inhibition of MAPK signaling suppresses the expression of core genes and impairs mycelial biomass accumulation, confirming that intact MAPK pathway activity may contribute to the low-temperature acclimatization and vegetative growth of *S. commune* 20R-7-F01.

### 3.6. cAMP Signaling Pathway Involved in Low-Temperature Stress

**Figure 6:**
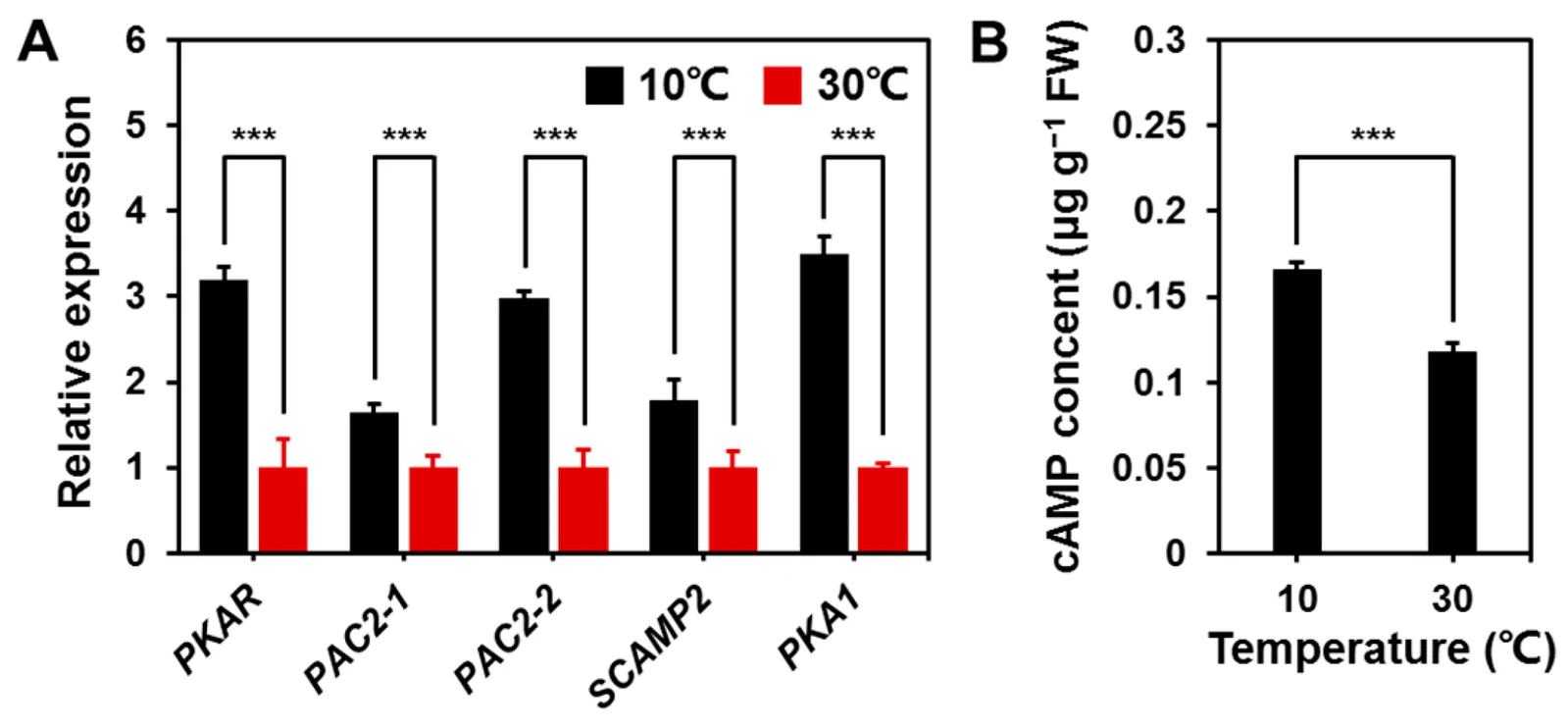
Effects of low temperature on the expression of genes associated with the cAMP signaling pathway (**A**) and intracellular cAMP content in mycelia (**B**). Genes involved in the 3′-5′-cyclic adenosine monophosphate (cAMP) signaling pathway: *PKAR*, *PAC2-1*, *PAC2-2*, *SCAMP2*, and *PKA1*. Mycelia were cultured in 150 mL of liquid MM at 10 °C or 30 °C for 96 h. μg g^−1^ FW represents the cAMP content per g of fresh weight. Values are presented as mean ± S.E (*n* = 3). Asterisk (*) indicates a significant difference compared to the 30 °C culture group. *** *p* < 0.001.

To elucidate whether the 3′-5′-cyclic adenosine monophosphate (cAMP) signaling cascade is involved in the low-temperature response of *S. commune* 20R-7-F01, we compared the transcriptional levels of five core genes related to the cAMP pathway in the 10 °C and 30 °C treatment groups. All detected genes showed detectable upregulation of transcription under low-temperature stress, with significant differences between genes (Figure 6). All five selected cAMP related genes showed higher transcript levels at 10 °C than at 30 °C, although the magnitude of the response differed among genes (Figure 6A).

At the same time, we measured the cAMP content in mycelial samples. As shown in Figure 6B, the cAMP content in the 10 °C treatment group (0.17 μg g^−1^ FW) was significantly higher than that in the 30 °C treatment group (0.12 μg g^−1^ FW), indicating that low temperature significantly promotes cAMP accumulation. Together, these transcriptional changes and elevated cAMP levels suggest that cAMP-associated responses may participate in the low-temperature response of *S. commune* 20R-7-F01.

### 3.7. Ca^2+^ Signaling Regulates Mycelial Integrity

**Figure 7:**
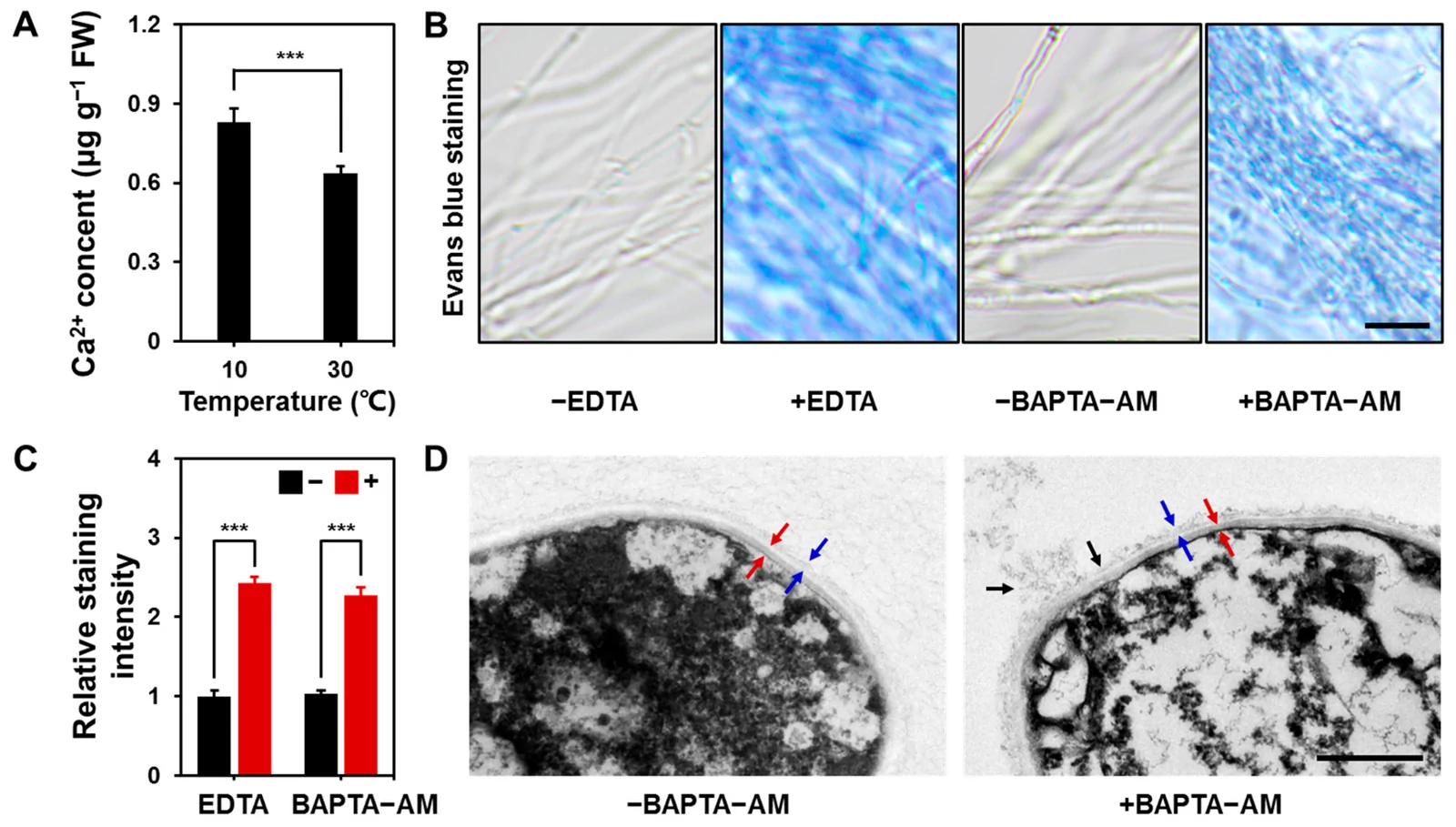
Low temperature promotes an increase in Ca^2+^ content within the mycelium of *S. commune* 20R-7-F01, whereas Ca^2+^ depletion disrupts cellular integrity. (**A**) Quantitative analysis of mycelial Ca^2+^ content under 10 °C and 30 °C treatments. Mycelia were cultured in 150 mL of liquid MM at 10 °C or 30 °C for 96 h. μg g^−1^ FW represents the Ca^2+^ content per g of fresh weight. Values are presented as mean ± S.E (*n* = 3). Asterisk (*) indicates a significant difference compared to the 30 °C culture group. *** *p* < 0.001. Bar = 10 μm. (**B**) Evans blue staining to detect cell membrane integrity of hyphae cultured at 10 °C; the experiment was conducted across four groups, with and without the addition of Ca^2+^ chelators (-EDTA/-BAPTA-AM and +EDTA/+BAPTA-AM). Enhanced blue staining indicates impaired cell membrane permeability. (**C**) Relative staining intensity of Evans blue stained images (relative to the -EDTA treatment). Values are presented as mean ± S.E (*n* = 3). Asterisk (*) indicates a significant difference compared to the culture group without calcium ion chelators (-EDTA/-BAPTA-AM). *** *p* < 0.001. (**D**) Effect of Ca^2+^ content on cell wall integrity under 10 °C treatment. Red arrows indicate the inner cell wall, blue arrows indicate the outer cell wall, and black arrows indicate cell wall damage. Mycelia were cultured at 10 °C for 96 h in 150 mL of liquid MM medium containing or lacking 20 μM BAPTA-AM. Bar = 500 nm.

Ca^2+^ is crucial for maintaining cellular integrity, given its role as an important second messenger. Therefore, we investigated the Ca^2+^ content within mycelia under low-temperature stress. The results showed that the intracellular Ca^2+^ content at 10 °C was 30.61% higher than that at 30 °C (Figure 7A), indicating that low-temperature stress promoted the increase in Ca^2+^ content in mycelia.

Evans blue staining further verified the dependence of cell membrane integrity on Ca^2+^ under low-temperature conditions (Figure 7B,C). At 10 °C, untreated mycelia (-EDTA) showed almost no blue staining, indicating that the cell membrane structure was intact and hindered the penetration of Evans blue. Conversely, significant blue staining was observed in mycelia cultured with exogenous EDTA (+EDTA). This significant staining signal reflects the damage and altered permeability of the fungal cell membrane after Ca^2+^ depletion. To further confirm that the cell membrane damage observed was specifically due to Ca^2+^ depletion rather than a nonspecific effect of EDTA, we treated mycelia with BAPTA-AM (Ca^2+^ specific chelating agent). The results showed that the BAPTA-AM treatment produced effects completely consistent with those of EDTA, with markedly increased Evans blue staining in mycelia under low-temperature conditions (Figure 7B,C). This consistency strongly supports that the loss of the cell membrane integrity is directly attributable to Ca^2+^ depletion.

To further elucidate the protective role of Ca^2+^ in maintaining cell wall integrity, we examined the cell walls using transmission electron microscopy (TEM) (Figure 7D). The results showed that under low-temperature stress, hyphae not treated with BAPTA-AM (-BAPTA-AM) exhibited intact cell wall structures. However, when intracellular Ca^2+^ was chelated (+BAPTA-AM), the hyphal cell walls displayed evident localized damage and even rupture (indicated by black arrows). These ultrastructural observations visually demonstrate that Ca^2+^ depletion under low-temperature stress leads to severe localized structural damage to the cell wall, thereby further confirming the critical role of Ca^2+^ in maintaining cell wall integrity. In summary, these results indicate that low-temperature stress induces an increase in mycelial Ca^2+^ content, and that intact Ca^2+^ signaling is crucial for maintaining cell structure integrity.

## 4. Discussion

Temperature is a major environmental factor regulating fungal growth, development, and reproduction. Temperature directly influences the rates of enzymatic reactions, metabolic flux, membrane properties, and intracellular transport processes in fungi, thereby affecting energy production and macromolecule synthesis [50,51,52]. In the present study, low temperature markedly inhibited mycelial growth and biomass accumulation and delayed fruiting body formation and basidiospore germination in *Schizophyllum commune* 20R-7-F01 (Figure 1 and Figure 2). These effects are consistent with previous observations in *Pleurotus ostreatus*, in which suboptimal temperatures reduced mycelial growth and delayed reproductive development [53]. Thus, the growth inhibition observed here is likely to result from the combined effects of reduced biochemical reaction rates and subsequent cellular stress responses. In particular, decreased enzyme activity at low temperature may limit nutrient utilization, energy production, and biosynthetic processes, providing a fundamental explanation for the slower vegetative and reproductive development.

The delayed reproductive development further suggests that temperature affects not only biomass accumulation but also developmental transitions. Primordium formation and fruiting body maturation are associated with cellular differentiation, metabolism, and tissue organization. A reduction in metabolic activity under low temperature may therefore slow the progression of these processes. The prolonged basidiospore germination time observed in this study further indicates that reproductive cells are also sensitive to temperature-dependent reductions in cellular activity (Figure 2). Similar temperature-dependent effects on mushroom fructification have been reported previously [53,54]. Therefore, the developmental delay observed in *S. commune* 20R-7-F01 may reflect the cumulative consequences of reduced metabolic efficiency rather than the regulation of a single developmental pathway.

Low temperature also compromises cell integrity, particularly at the level of the cell wall. The fungal cell wall provides mechanical support and protects cells against environmental stress. In our study, increased chitin content and induction of *CHS* expression under low-temperature conditions indicate that cell wall integrity may have been compromised (Figure 3). Similar increases in chitin synthesis have been associated with maintenance of cell integrity under stress in other fungi [55,56]. This phenomenon may represent a compensatory response that helps maintain structural stability when cellular growth is restricted. However, this study did not directly verify whether this cell wall maintenance strategy comes at the expense of growth. Therefore, the observed changes suggest a possible trade-off between cell wall maintenance and fungal growth that warrants further investigation.

Low-temperature exposure also disturbed cellular redox homeostasis. Short-term low-temperature treatment increased ROS and MDA levels while decreasing GSH/GSSG, GSH-Px, SOD, and T-AOC, indicating an early oxidative imbalance (Figure 4). Low temperature can disturb metabolic and respiratory processes, resulting in transient ROS accumulation [51,52]. However, prolonged low-temperature exposure reduced ROS and MDA levels and restored antioxidant capacity, suggesting that *S. commune* 20R-7-F01 gradually acclimated to low-temperature conditions. This transition indicates that ROS may function not only as damaging molecules but also as signals that initiate antioxidant and protective responses. The dynamic regulation of ROS therefore appears to be an important component of low-temperature acclimation in *S. commune* 20R-7-F01.

Signaling pathways may further be associated with these cellular responses. MAPK pathways are conserved regulators of fungal growth, cell wall integrity, and environmental stress responses. Interference of MAPK signaling with U0126 reduced mycelial biomass and suppressed the expression of several MAPK-related genes under low-temperature conditions (Figure 5), supporting an important role for MAPK signaling in maintaining growth during low-temperature stress. Similar functions of MAPK pathways have been reported in *Aspergillus fumigatus* and *Magnaporthe grisea* [57,58]. Our results extend this concept to mushroom-forming fungi and suggest that MAPK signaling participates in the cellular adjustment of *S. commune* 20R-7-F01 to low temperature.

The 3′-5′-cyclic adenosine monophosphate (cAMP) pathway also appeared to participate in low-temperature adaptation. All examined cAMP-related genes showed higher transcript levels, and mycelial cAMP content increased, under low-temperature conditions (Figure 6). The cAMP/PKA pathway regulates fungal growth, differentiation, metabolism, and stress responses and may therefore connect environmental signals with cellular reprogramming [59]. Its induction under low-temperature conditions suggests that cAMP-associated signaling may contribute to maintaining cellular activity and developmental processes when metabolic rates are reduced. However, because our evidence is primarily based on transcriptional changes and cAMP levels, further research is required to determine intracellular PKA activity and establish the functional role of this pathway.

Ca^2+^ signaling provides another potential mechanism linking low-temperature perception with cellular protection. Mycelial Ca^2+^ content increased by 30.61% under low-temperature conditions, whereas Ca^2+^ depletion resulted in impairment of cellular integrity (Figure 7). Ca^2+^ is an important second messenger involved in fungal stress responses and cell wall regulation [60]. The increased Ca^2+^ content observed here is consistent with a possible role of Ca^2+^ signaling in maintaining cellular integrity under low-temperature stress. However, whether Ca^2+^ acts upstream of MAPK or cAMP signaling, and whether these pathways interact under low-temperature stress, remains to be determined. Likewise, the effects of Ca^2+^ depletion on chitin content and *CHS* expression were not directly examined in this study. Together with the MAPK and cAMP responses, Ca^2+^ signaling may be part of a broader regulatory network associated with cellular adaptation to low temperature, although the relationships among these pathways require further experimental validation.

Collectively, our results indicate that low-temperature substantially restricts the growth and reproduction of *S. commune* 20R-7-F01, while simultaneously inducing multiple physiological responses. Low-temperature treatment was associated with cellular integrity, antioxidant responses, and changes in MAPK, cAMP, and Ca^2+^-related responses. Importantly, the present study addresses only one component of this complex process. Low temperature may also affect numerous metabolic enzymes, energy production, membrane lipid composition, protein synthesis, and nutrient transport, none of which were directly examined here. Therefore, our findings should be interpreted as evidence for the involvement of cellular integrity, redox, and signaling regulation in low-temperature adaptation rather than as a comprehensive explanation of temperature-dependent growth inhibition. Integrating enzyme activity assays with transcriptomic, proteomic, and metabolomic analyses will be necessary to further clarify the mechanisms underlying low-temperature regulated growth and development in mushroom-forming fungi.

Several limitations should be considered when interpreting the results of this study. First, the strain examined was isolated from a deep-sea environment; its physiological, metabolic, and ecological characteristics may differ from those of isolates originating from other environments. Different fungal strains exhibit varying capacities to adapt to low temperatures; consequently, the physiological responses and underlying mechanisms observed in this specific strain may not necessarily be representative of strains from other environments. Furthermore, fungal responses to temperature depend on the species and developmental stage; for certain fungi that produce large fruiting bodies, a drop in temperature under conditions of adequate moisture, light, and ventilation may actually promote rather than delay specific developmental transitions. Therefore, the responses observed in the *S. commune* 20R-7-F01 should not be generalized to all basidiomycetes. Second, the study did not include multiple isolates of the same species, which limited the ability to assess intraspecific variation and distinguish between strain-specific and species-level characteristics. Third, this study is descriptive and hypothesis-generating; the pathway-related data reflect correlations rather than causal relationships, and causal validation through gene manipulation and biochemical analyses is still required. Fourth, the lack of proteomic and metabolomic data limits a comprehensive understanding of the downstream molecular events. Finally, the findings should be viewed as preliminary results intended to generate hypotheses rather than as definitive conclusions at the species level. Future comparative studies involving multiple strains from diverse geographical regions and environments will be conducted to determine the degree to which the observed characteristics are conserved within the species.

## Data Availability

The original contributions presented in this study are included in the article/Supplementary Materials. Further inquiries can be directed to the corresponding author.

## Supplementary Materials

The following supporting information can be downloaded at: https://www.mdpi.com/article/10.3390/jof12100760/s1, Table S1: Primers used in this study.

## Abbreviations

The following abbreviations are used in this manuscript:

*CHS*	Chitin synthase genes
MAPK	Mitogen-activated protein kinase
cAMP	3′-5′-cyclic adenosine monophosphate
ROS	Reactive oxygen species
GSH	Reduced glutathione
GSSG	Oxidized glutathione
GSH-Px	Glutathione peroxidase
MM	Minimal medium
RT-qPCR	Reverse transcription quantitative polymerase chain reaction
SPSS	Statistical package for social sciences
MDA	Malondialdehyde
SOD	Superoxide dismutase
T-AOC	Total antioxidant capacity
TEM	Transmission electron microscopy

## References

[1] Li D., Chu C., Zhao M., Hou S., Ji R., Liu C. (2025). Nitric oxide-mediated regulation of chitinase activity and cadmium sequestration in the response of *Schizophyllum commune* to cadmium stress. Microorganisms.

[2] Chu C., Li D., Gu L., Yang S., Liu C. (2025). Evidence for the existence of mating subtypes within the *Schizophyllum commune*: Mating behavior and genetic divergence. J. Fungi..

[3] Liu X., Huang X., Chu C., Xu H., Wang L., Xue Y., Muhammad Z., Inagaki F., Liu C. (2022). Genome, genetic evolution, and environmental adaptation mechanisms of *Schizophyllum commune* in deep subseafloor coal-bearing sediments. iScience.

[4] Zhang Y., Kong H., Fang Y., Nishinari K., Phillips G. (2013). Schizophyllan: A review on its structure, properties, bioactivities and recent developments. Bioact. Carbohydr. Diet. Fibre..

[5] Ohm R., De Jong J., Lugones L., Aerts A., Kothe E., Stajich J., de Vries R., Record E., Levasseur A., Baker S. (2010). Genome sequence of the model mushroom *Schizophyllum commune*. Nat. Biotechnol..

[6] Xiao W., Zhang J., Huang J., Xin C., Li M., Song Z. (2022). Response and regulatory mechanisms of heat resistance in pathogenic fungi. Appl. Microbiol. Biotechnol..

[7] Virágh M., Merényi Z., Csernetics Á., Földi C., Sahu N., Liu X., Hibbett D., Nagy L. (2022). Evolutionary morphogenesis of sexual fruiting bodies in basidiomycota: Toward a new evo-devo synthesis. Microbiol. Mol. Biol. Rev..

[8] Nowrousian M. (2022). The role of chromatin and transcriptional control in the formation of sexual fruiting bodies in fungi. Microbiol. Mol. Biol. Rev..

[9] Zhang X., Wang Z., Jiang C., Xu J. (2021). Regulation of biotic interactions and responses to abiotic stresses by MAP kinase pathways in plant pathogenic fungi. Stress Biol..

[10] Leng S., Liu C. (2026). Environmental adaptation strategies of deep-sea fungi. J. Fungi..

[11] Pavankumar T., Rai N., Pandey P., Vincent N. (2024). Helicases at work: The importance of nucleic acids unwinding under cold stress. DNA.

[12] Baskaran A., Kaari M., Venugopal G., Manikkam R., Joseph J., Bhaskar P. (2021). Anti freeze proteins (Afp): Properties, sources and applications-a review. Int. J. Biol. Macromol..

[13] Arai T., Fukami D., Hoshino T., Kondo H., Tsuda S. (2019). Ice-binding proteins from the fungus *Antarctomyces psychrotrophicus* possibly originate from two different bacteria through horizontal gene transfer. FEBS J..

[14] Weinstein R., Palm M., Johnstone K., Wynn-Williams D. (1997). Ecological and physiological characterization of *Humicola marvinii*, a new psychrophilic fungus from fellfield soils in the maritime Antarctic. Mycologia.

[15] Jennings D., Rose A., Tempest D. (1985). Polyol metabolism in fungi. Advances in Microbial Physiology.

[16] Fang W., Leger R.S. (2010). RNA binding proteins mediate the ability of a fungus to adapt to the cold. Environ. Microbiol..

[17] Duarte A., Dos Santos J., Vianna M., Vieira J., Mallagutti V., Inforsato F., Wentzel L., Lario L., Rodrigues A., Pagnocca F. (2018). Cold-adapted enzymes produced by fungi from terrestrial and marine Antarctic environments. Crit. Rev. Biotechnol..

[18] Saharova S., Ianutsevich E., Danilova O., Grum-Grzhimaylo O., Groza N., Tereshina V. (2025). Cold adaptation mechanisms of the psychrotolerant filamentous ascomycete *Psychrophilomyces antarcticus*. Braz. J. Microbiol..

[19] Kitamoto D., Yanagishita H., Endo A., Nakaiwa M., Nakane T., Akiya T. (2001). Remarkable antiagglomeration effect of a yeast biosurfactant, diacylmannosylerythritol, on ice-water slurry for cold thermal storage. Biotechnol. Prog..

[20] Perfumo A., Banat I., Marchant R. (2018). Going green and cold: Biosurfactants from low-temperature environments to biotechnology applications. Trends Biotechnol..

[21] Gow N., Lenardon M. (2023). Architecture of the dynamic fungal cell wall. Nat. Rev. Microbiol..

[22] Qin J., Zhao P., Ye Z., Sun L., Hu X., Zhang J. (2022). Chitin synthase genes are differentially required for growth, stress response, and virulence in *Verticillium dahliae*. J. Fungi..

[23] Robinson C. (2001). Cold adaptation in arctic and antarctic fungi. New Phytol..

[24] Butler M., Day A. (1998). Fungal melanins: A review. Can. J. Microbiol..

[25] Yoshimi A., Miyazawa K., Kawauchi M., Abe K. (2022). Cell wall integrity and its industrial applications in filamentous fungi. J. Fungi..

[26] Singh Y., Nair A., Verma P. (2021). Surviving the odds: From perception to survival of fungal phytopathogens under host-generated oxidative burst. Plant Commun..

[27] Cansado J., Soto T., Franco A., Vicente-Soler J., Madrid M. (2021). The fission yeast cell integrity pathway: A functional hub for cell survival upon stress and beyond. J. Fungi..

[28] Sun Z., Yu S., Wang C., Wang L. (2022). cAMP signalling pathway in biocontrol fungi. Curr. Issues Mol. Biol..

[29] Roy A., Kumar A., Baruah D., Tamuli R. (2021). Calcium signaling is involved in diverse cellular processes in fungi. Mycology.

[30] Stępień Ł., Lalak-Kańczugowska J. (2021). Signaling pathways involved in virulence and stress response of plant-pathogenic *Fusarium* species. Fungal Biol. Rev..

[31] Liu C., Huang X., Xie T., Duan N., Xue Y., Zhao T., Lever M., Hinrichs K., Inagaki F. (2017). Exploration of cultivable fungal communities in deep coal-bearing sediments from ~1.3 to 2.5 km below the ocean floor. Environ. Microbiol..

[32] Zain Ul Arifeen M., Ma Z., Wu S., Liu J., Xue Y., Liu C. (2021). Effect of oxygen concentrations and branched-chain amino acids on the growth and development of sub-seafloor fungus, *Schizophyllum commune* 20R-7-F01. Environ. Microbiol..

[33] Li D., Chu C., Zhao M., Hou S., Liu C. (2025). The role of nitric oxide in the growth and development of *Schizophyllum commune* under anaerobic conditions. Microorganisms.

[34] Tkaczuk C., Majchrowska-Safaryan A. (2023). Temperature requirements for the colony growth and conidial germination of selected isolates of entomopathogenic fungi of the *Cordyceps* and *Paecilomyces genera*. Agriculture.

[35] Ma D., Ji Y., Yang F., Liu W., Wan Z., Li R. (2013). Effects of U0126 on growth and activation of mitogen-activated protein kinases in *Aspergillus fumigatus*. Chin. Med. J..

[36] Liu P., Gong J., Ding X., Jiang Y., Chen G., Li B., Weng Q., Chen Q. (2016). The L-type Ca^2+^ channel blocker nifedipine inhibits mycelial growth, sporulation, and virulence of *Phytophthora capsici*. Front. Microbiol..

[37] Westman J., Walpole G., Kasper L., Xue B., Elshafee O., Hube B., Grinstein S. (2020). Lysosome fusion maintains phagosome integrity during fungal infection. Cell Host Microbe.

[38] d’Enfert C. (1997). Fungal apore germination: Insights from the molecular genetics of *Aspergillus nidulans* and *Neurospora crassa*. Fungal Genet. Biol..

[39] Smith R., Gilkerson E. (1979). Quantitation of glycosaminoglycan hexosamine using 3-methyl-2-benzothiazolone hydrazone hydrochloride. Anal. Biochem..

[40] Eruslanov E., Kusmartsev S., Armstrong D. (2009). Identification of ROS using oxidized DCFDA and flow-cytometry. Advanced Protocols in Oxidative Stress II.

[41] Ohkawa H., Ohishi N., Yagi K. (1978). Reaction of linoleic acid hydroperoxide with thiobarbituric acid. J. Lipid Res..

[42] Alpert A., Gilbert H. (1985). Detection of oxidized and reduced glutathione with a recycling postcolumn reaction. Anal. Biochem..

[43] Chen L., Tian X., Yang B. (1990). A study on the inhibition of rat myocardium glutathione peroxidase and glutathione reductase by moniliformin. Mycopathologia.

[44] Benzie I., Strain J. (1996). The ferric reducing ability of plasma (FRAP) as a measure of “antioxidant power”: The FRAP assay. Anal. Biochem..

[45] Masayasu M., Hiroshi Y. (1979). A simplified assay method of superoxide dismutase activity for clinical use. Clin. Chim. Acta.

[46] Bradford M. (1976). A rapid and sensitive method for the quantitation of microgram quantities of protein utilizing the principle of protein-dye binding. Anal. Biochem..

[47] Pfaffl M. (2001). A new mathematical model for relative quantification in real-time RT-PCR. Nucleic Acids Res..

[48] Livak K., Schmittgen T. (2001). Analysis of relative gene expression data using real-time quantitative PCR and the 2^−ΔΔCT^ method. Methods.

[49] Di Ciaccio L., Catalano A., López P., Rojas D., Cristos D., Fortunato R., Salvat A. (2020). In vitro antifungal activity of *Peltophorum dubium* (Spreng.) taub. extracts against *Aspergillus flavus*. Plants.

[50] Knapp B., Huang K. (2022). The effects of temperature on cellular physiology. Annu. Rev. Biophys..

[51] Shen L., Zhang S., Chen G. (2021). Regulated strategies of cold-adapted microorganisms in response to cold: A review. Environ. Sci. Pollut. Res..

[52] Yusof N., Hashim N., Bharudin I. (2021). Cold adaptation strategies and the potential of psychrophilic enzymes from the antarctic yeast, *Glaciozyma antarctica* PI12. J. Fungi..

[53] Hu Y., Xue F., Chen Y., Qi Y., Zhu W., Wang F., Wen Q., Shen J. (2023). Effects and mechanism of the mycelial culture temperature on the growth and development of *Pleurotus ostreatus* (Jacq.) P. Kumm. Horticulturae.

[54] Sakamoto Y. (2018). Influences of environmental factors on fruiting body induction, development and maturation in mushroom-forming fungi. Fungal Biol. Rev..

[55] Kleijburg F., Schunselaar E., Safeer A., Bishoyi A., Baldus M., Wösten H. (2025). Functions of the cell wall polysaccharide schizophyllan during vegetative growth of *Schizophyllum commune*. Cell Surf..

[56] Yuan Z., Ge Z., Fu Q., Wang F., Wang Q., Shi X., Wang B. (2024). Investigation of cold-resistance mechanisms in cryophylactic yeast *Metschnikowia pulcherrima* based on comparative transcriptome analysis. Front. Microbiol..

[57] Jain R., Valiante V., Remme N., Docimo T., Heinekamp T., Hertweck C., Gershenzon J., Haas H., Brakhage A. (2011). The MAP kinase MpkA controls cell wall integrity, oxidative stress response, gliotoxin production and iron adaptation in *Aspergillus fumigatus*. Mol. Microbiol..

[58] Zhao X., Kim Y., Park G., Xu J. (2005). A mitogen-activated protein kinase cascade regulating infection-related morphogenesis in *Magnaporthe grisea*. Plant Cell.

[59] Bahn Y., Xue C., Idnurm A., Rutherford J., Heitman J., E Cardenas M. (2007). Sensing the environment: Lessons from fungi. Nat. Rev. Microbiol..

[60] Bakar N., Karsani S., Alias S. (2020). Fungal survival under temperature stress: A proteomic perspective. PeerJ.

