# Supplementary material for: Calcium-Associated Cellular Integrity and MAPK/cAMP Signaling Are Associated with Low-Temperature Acclimation in Schizophyllum commune 20R-7-F01
Source: J Fungi (Basel). 2026 Oct 9;12(10):760. doi: 10.3390/jof12100760 (PMC13705983; doi:10.3390/jof12100760)
Supplement: Supplementary file 1 [file jof-12-00760-s001.zip › Supplementary Information.pdf]

Supplementary Information for:

Calcium-Associated Cellular Integrity and MAPK/cAMP Signaling Are Associated with Low-Temperature Acclimation in *Schizophyllum commune* 20R-7-F01

Shuang Leng [1,†], Dongxu Li [1,†], Chen Chu [1,2], Changhong Liu[1,*]

1 State Key Laboratory of Pharmaceutical Biotechnology, School of Life Sciences, Nanjing University,Nanjing 210023, China; lengshuang@nju.edu.cn (S.L.); laolang_2012@163.com (D.L.); mg1930112@smail.nju.edu.cn (C.C.).

2 First Clinical Medical College, Nanjing University of Chinese Medicine, Nanjing 210023, China.

* Correspondence: Changhong Liu (chliu@nju.edu.cn).

† These authors have contributed equally to this work.

Table S1. Primers used in this study

Primer	Sequence (5'-3')	Application
Actin-F	TGGTATCCTCACGTTGAAGTA	Real-time RT-PCR (internal control)
Actin-R	GTGTGGTGCCAGATCTT	Real-time RT-PCR (internal control)
CHS1-F	CTCCTATGTGCTGGTGTC	Real-time RT-PCR
CHS1-R	CGTTCGTGGTGTTGTAGA	Real-time RT-PCR
CHS2-F	CCTGCTACAACACCTACAA	Real-time RT-PCR
CHS2-R	ATGGCTGCTCTGATACGA	Real-time RT-PCR
CHS3-1-F	TCAAGGAGCGAAGTTCAAG	Real-time RT-PCR
CHS3-1-R	AGGCGTAGATGTTCAAGAC	Real-time RT-PCR
CHS3-2-F	TCATCTCCATCGGCTTCA	Real-time RT-PCR
CHS3-2-R	ATGTCGTTACTCCAGTTCTC	Real-time RT-PCR
CHS4-F	GCAGGAACAAGACATCAAC	Real-time RT-PCR
CHS4-R	TACGGAGGCGAACATACT	Real-time RT-PCR
CHS6-F	TCTACCTCATCTACCTCATCA	Real-time RT-PCR
CHS6-R	ACCTTCTTATCCTTGCCTTC	Real-time RT-PCR
CHS7-F	CAGCACGACGATGACTAT	Real-time RT-PCR
CHS7-R	TAACACCTCGCTTGATGG	Real-time RT-PCR
CHS8-F	ATCACGAAGAAGATGGAGAG	Real-time RT-PCR
CHS8-R	CAGGATGTAGGTAGAGAAGAG	Real-time RT-PCR
MAPK-F	GACGAGTTCTACGCCATC	Real-time RT-PCR
MAPK-R	GCTGCTCACGACTTATGT	Real-time RT-PCR
MTS50-F	TACGAGCAGCAGATCAGA	Real-time RT-PCR
MTS50-R	TGGTGGCACATAATCATCC	Real-time RT-PCR
MKP2-F	ACCTTCTAACCTCCTCTTGA	Real-time RT-PCR
MKP2-R	TGGTGGTGGTAGTCTCTG	Real-time RT-PCR
MPS1-F	GTCTGGTCTATTGGCTGTAT	Real-time RT-PCR
MPS1-R	CGAGGTAGTGGAGAATCTG	Real-time RT-PCR
HOG1-F	ACGAGAATTGCGACTTGA	Real-time RT-PCR
HOG1-R	GATGATAGAGAACTGGTTGAC	Real-time RT-PCR
MCK1-F	CATCTGAGTCTGCTCTCC	Real-time RT-PCR
MCK1-R	GGATCTCCACTTGCTTGA	Real-time RT-PCR
MST11-F	GCTCATCACGACACATCT	Real-time RT-PCR
MST11-R	GGTTGCCTGTTCTTCTCC	Real-time RT-PCR
PKAR-F	CTTCGCATCGCTACTTCT	Real-time RT-PCR
PKAR-R	GAGGTCGTCAATGAGTGTT	Real-time RT-PCR
PAC2-1-F	AACCTACCTGTACCAATATCC	Real-time RT-PCR
PAC2-1-R	CATTCACTTCGACGTTCTG	Real-time RT-PCR
PAC2-2-F	CGTACTCTGTCTTCATCAAC	Real-time RT-PCR
PAC2-2-R	ATGTCGTCTATCGTCTGTAG	Real-time RT-PCR
SCAMP2-F	CTCATCTTCCACTCCATCAA	Real-time RT-PCR
SCAMP2-R	GCCTGCTCCTTCATATACC	Real-time RT-PCR
PKA1-F	CAAGTGGAGCATGTGAAC	Real-time RT-PCR

PKA1-R	CGGAGGAAGTATGTGGAAT	Real-time RT-PCR
